# Mapping the gaps: Multi-disciplinary chronic pain service provision for cancer-related pain across England

**DOI:** 10.1177/20494637261465816

**Published:** 2026-07-17

**Authors:** Julie Armoogum, Alison Llewellyn, Fiona Cramp, Alice Berry, Candida McCabe

**Affiliations:** 1 1981 School of Health and Social Wellbeing, University of the West of England, Bristol, UK; 2 58602 Centre for Education and Research, Dorothy House Hospice Care, Bradford on Avon, UK

**Keywords:** pain, cancer, cancer-related pain, late effects, cancer survivor, services, access

## Abstract

**Introduction:**

Prevalence rates for cancer-related pain are 40-50%. Provision of multi-disciplinary chronic pain services for those with cancer-related pain is poorly understood.

**Aim:**

To identify provision of multi-disciplinary chronic pain services for those with cancer-related pain across England.

**Methods:**

Acute/Hospital and Community NHS Trusts in England (*n* = 190) were sent Freedom of Information requests asking if a multi-disciplinary chronic pain service was provided by the Trust, and numbers of people seen in previous 24 months with (a) tumour-related pain and (b) cancer-related late effects pain or pain caused by cancer treatments. Demographic information was requested. If no response was received within 10 weeks, a follow up request was sent. Quantitative data were analysed using descriptive statistics.

**Results:**

In total, 171 NHS Trusts responded. Almost half, (*n* = 74, 43.3%) reported providing a multi-disciplinary chronic pain service. Of these, 17 (23.0%) were able to provide attendance data for people with tumour-related pain and eight (10.8%) with late effects pain. Demographic information could be provided by seven (9.5%) Trusts for those with tumour-related pain and four (5.4%) for late effects pain. The main reason Trusts could not provide demographic information was because such data were not routinely collected.

**Conclusions:**

People with cancer-related pain appear to have limited access to multi-disciplinary chronic pain support in England. However, lack of standardised methods of coding and recording cancer-related pain as reasons for attending multi-disciplinary chronic pain services mean reporting accurate figures is challenging. It is essential that there is an accurate reporting system so the scale of cancer-related pain can be understood to help guide the provision and development of services, education and research.

## Introduction

Cancer incidence continues to rise globally, and in the UK, one in two individuals is expected to receive a cancer diagnosis during their lifetime.^1–3^ While survival rates have improved, many people experience long-term consequences of cancer and its treatment, with pain frequently cited as a major concern and unmet need.^4^ Pain is reported by approximately 40% of cancer survivors across different tumour types.^5–10^ The International Association for the Study of Pain (IASP) defines chronic cancer-related pain as pain caused by the primary cancer, metastases, or its treatment.^11^

In England, healthcare is predominately provided by the National Health Service (NHS) and is funded by the government, free at the point of need for patients.^12^ The NHS is divided into Trusts that act as healthcare providers for hospital services, community services and/or other aspects of patient care such as ambulance services.^13^ Some private healthcare providers offer pain management services. This study does not include private healthcare pain management provision. Current UK guidance recommends that all healthcare professionals should recognise and screen for pain, and that individuals with persistent pain should have access to multi-disciplinary specialist pain management services.^14,15^ This includes people living with cancer who may benefit from joint management with palliative care, as well as cancer survivors experiencing chronic pain following treatment. However, the epidemiology and demographic characteristics of people attending chronic pain services in the UK remain poorly understood,^14,16^ and attendance rates for those with chronic cancer-related pain are unknown. Globally, cancer survivors report difficulties accessing pain support^17–20^ and healthcare professionals describe limited and inconsistent services for managing cancer-related pain.^21,22^

To address these gaps, this study aimed to identify the provision of multi-disciplinary chronic pain services for individuals with cancer-related pain across England. Specifically, the objectives were:

1. To identify multi-disciplinary chronic pain services in England that accept people with cancer-related pain.
2. To establish the demographic characteristics of individuals with chronic cancer-related pain seen in these services.

The reporting of this study follows the REporting of studies Conducted using Observational Routinely collected health Data (RECORD) statement.^23^

## Methods

### Study development

Freedom of Information (FOI) requests were considered an appropriate approach because they enable data to be gained from a wide range of NHS Trusts.^24–27^ The Freedom of Information Act^28^ was passed in 2000 in England and enabled right of access to information held by public authorities, including the NHS, with an aim to promote greater openness and accountability. FOI requests have been utilised successfully to establish care pathways and provision within the NHS^25,29,30^ and Macgregor and colleagues (2026) adopted this approach to map chronic pain services in Scotland, UK.^31^ However, obtaining data from FOI is more challenging than people may assume and therefore researchers must consider and plan for how to access the data and ensure they ask clear and unambiguous questions.^32^ As Walby and Luscombe argue, dealing with data on this scale is no less difficult than traditional methods of data collection and requires systematic methods of data collection, storage and analysis.^32^ FOI requests can be refused if it would cost too much or take too much staff time to deal with the request, or it the request is vexatious or the information is already publicly available.^33^ To minimise this risk for the current study, firstly, the FOI disclosure log for each Trust was reviewed to ensure the information was not already available and secondly, we consulted with stakeholders to ensure the FOI request questions were clear. We conducted a series of stakeholder consultation events to refine the FOI questions with a total of 28 stakeholders: (1) an online presentation and discussion via MS Teams with 17 researchers, academics and healthcare professionals with an interest in supportive, palliative and end of life care (2) an in person discussion with a General Practitioner (GP) (3) in person discussions with two NHS employees who have experience completing FOI requests but do not work in pain services (3) online discussion via MS Teams and written feedback from a nurse, clinician, administrator and manager from the national NHS Pain-related Complex Cancer Late Effects Rehabilitation Service (CCLERS) and two nurses working in NHS chronic pain services with experience of completing FOI requests, and (4) two people with lived experience of cancer-related pain. Discussions in the stakeholder events highlighted practical and philosophical concerns with FOI requests. Practically, the importance of asking unambiguous questions that elicit the correct information was raised. Philosophical concerns were also raised, such as the negative view organisations can have towards FOI request. This is supported in the literature as organisations can consider FOI requests to be intrusive, which can lead to undue secrecy and provision of minimal information.^26^ Actions taken following the stakeholder consultations included amending the questions to provide clarity about cancer-related pain including separating ‘cancer tumour-related pain’ and ‘late effects pain’. The researcher’s name, contact details and purpose of the research was included in an introductory email alongside the FOI request (Supplemental File 1). The final questions included in the FOI request are outlined in box 1.

**Box 1. FOI request questions**

(1a) Is a multi-disciplinary chronic pain service provided by the Trust? (if yes, please continue to question 2, if no please answer question 1b)

(1b) Where is chronic pain being managed in your organisation for people with:

(i) Cancer tumour-related pain (i.e. people with a current oncological or haematological cancer diagnosis who are experiencing pain)
(ii) Cancer-related late effects pain or chronic pain caused by cancer treatments (i.e. pain caused by chemotherapy, radiotherapy, immunotherapy, hormone therapy or surgery)

(2) Is the multi-disciplinary chronic pain service based in an acute hospital or a community setting? (please expand if needed)

(3) What is the current waiting time from referral to first appointment at the multi-disciplinary chronic pain service?

(4) Since October 2022, how many people did the multi-disciplinary chronic pain service see with:

(i) Cancer tumour-related pain (i.e. people with a current oncological or haematological cancer diagnosis who are experiencing pain)
(ii) Cancer-related late effects pain or chronic pain caused by cancer treatments (i.e. pain caused by chemotherapy, radiotherapy, immunotherapy, hormone therapy or surgery)

(5) Can you please provide demographic information (% gender, age range and % ethnicity) for those seen in the multi-disciplinary chronic pain service with:

(i) Cancer tumour-related pain (i.e. people with a current oncological or haematological cancer diagnosis who are experiencing pain)
Gender:
Age range:
Ethnicity:
(ii) Cancer-related late effects pain or chronic pain caused by cancer treatments (i.e. pain caused by chemotherapy, radiotherapy, immunotherapy, hormone therapy or surgery)
Gender:
Age range:
Ethnicity:

(6) If you cannot answer question 5, can you please explain why?

Thank you for your time and information, it is much appreciated.

### Recruitment

In October 2024, all NHS Trusts in England were identified from the online NHS England Trust Directory. Child, ambulance and mental health NHS trusts were excluded. The website for each of the included Trusts (n = 190) was searched to identify (a) the FOI disclosure log to ensure a previous similar request had not been submitted, and (b) the process for submitting a FOI request. FOI requests were submitted to 190 Trusts according to their individual process: 170 via introductory email from the lead author to a specific FOI email address with the FOI request form as an attachment, 18 via a form embedded into the Trust website, and two required set up of a password protected FOI account prior to submission of the request. A follow up request was sent in January 2025 to non-responders.

### Data collection

Completed FOI responses, most commonly in PDF format, were sent from NHS Trusts to the lead author via email. Some Trusts sent the information via a password protected account with a corresponding email to the lead author. Data were inputted into an Excel database with each Trust numbered at random. Data collection took place over 4 months.

### Analysis

Quantitative data were analysed in Excel using descriptive statistics

## Results

**Figure 1.**
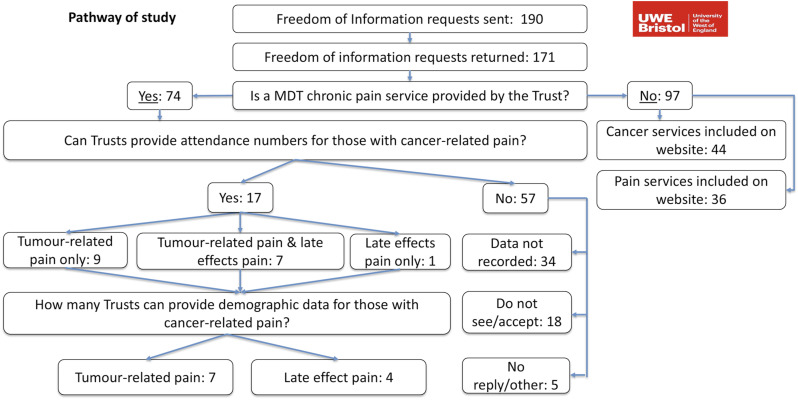
Provision of multi-disciplinary chronic pain services for those with cancer-related pain across England.

Within the 4-month data collection period (October 2024 and January 2025), 171 NHS Trusts responded (90% response rate). Almost half, (*n* = 74, 43.3%) reported they provided a multi-disciplinary chronic pain service. Of these, 17 (23.0%) were able to provide attendance data for those with cancer-related pain, nine for tumour-related pain only, seven for tumour-related pain and late effects pain and one for late effects pain only (this service was for late effects pain only) and the remainder stated they were unable to provide this information. Under the remit of FOI legislation and within the context of this study, only seven (9.5%) were able to provide demographic information for those with tumour-related pain and four (5.4%) for late effects pain. Of the remaining 57 Trusts who had a multi-disciplinary chronic pain service, 60.0% (*n* = 34) reported they were not able to provide the attendance numbers for those with tumour-related pain and 77.2% (*n* = 44) for late effects pain nor demographic information for those with tumour-related pain (n = 46, 81.0%) nor late effects pain (*n* = 49, 86.0%) as it was not routinely collected. Almost a third (*n* = 18, 31.6%) explicitly reported the service did not see people with tumour-related pain and 12 (21.1%) did not see those with late effects pain (Figure 1). In response to the question about location of multi-disciplinary chronic pain services, 33 Trusts (44.6%) stated the service was in the hospital, 18 (24.3%) explained the service was in the hospital and community, 11 (14.9%) were in the community only and 12 (16.2%) did not reply. Waiting list time was provided by 48 Trusts and was a mean of 28.9 weeks (range 3-65).

### Access to multi-disciplinary chronic pain services for those with tumour-related chronic pain

Of the 16 multi-disciplinary chronic pain services that saw people with tumour-related pain, 14 provided attendance data and the range of people seen over 2-year period with tumour-related pain was 0-1645 patients, with a median of 21.5. Two specialist cancer Trusts saw the most people (1645 and 1406). The mean attendance rate over 2 years across the remaining multi-disciplinary chronic pain services was 39.2 patients.

### Access to multi-disciplinary chronic pain services for those with cancer-related late effects pain

Of the eight multi-disciplinary chronic pain services that saw people with cancer-related late effects pain, the range for attendance over 2 years was 0-1104 patients with a median of 8.5. Two specialist cancer Trusts saw the most (1104 and 299) and one service was exclusively for late effects pain. The mean across the remaining multi-disciplinary chronic pain services was 18.5 patients over 2 years.

### Where people with cancer-related pain were seen if Trust does not have multi-disciplinary chronic pain service

Over half of the 171 NHS Trusts that responded (n = 97, 56.7%) reported they did not provide a multi-disciplinary chronic pain service and said those with cancer-related pain were seen by cancer services, GPs, palliative care, community services, pain consultants or hospices. Of the 97 NHS Trusts that did not provide a multi-disciplinary chronic pain service, 44 (45.4%) included cancer service provision, and 36 (37.0%) included pain service provision, on their website (Figure 1).

## Discussion

This study aimed to identify the provision of multi-disciplinary chronic pain services for individuals with cancer-related pain across England and establish the demographic characteristics of those with chronic cancer-related pain seen in these services. We found that less than a quarter of NHS Trusts who had a multi-disciplinary chronic service were able to provide attendance data on how many people with cancer-related pain attended their services. For the three quarters of multi-disciplinary chronic pain services who could not provide attendance rates for those with cancer-related pain (n = 57), the main reason cited was that the data were not routinely collected. Thus, some services may have actually seen people with cancer-related pain but were unable to report it. Inadequate reporting and recording of where people with cancer-related pain are being seen presents some challenges. Firstly, it makes it hard to identify and describe if people with cancer-related pain are being supported by multi-disciplinary chronic pain services and secondly, it makes it difficult to understand the care pathways of those with cancer-related pain.

The International Association for the Study of Pain (IASP) have produced definitions to help clinicians diagnose and record the nuances and specification of cancer-related pain^11^ but healthcare professionals, service managers, researchers, commissioners and policy makers need to be able to track such patients within the healthcare system. One method is clinical coding, a process by which healthcare data, including diagnoses, investigations, and treatments, are translated into codes. These codes are then utilised to help organisations better understand the activities they undertake, both in terms of the types of patients cared for and the treatments delivered.^34^ It is essential that clinical coding accurately reflects the patient population so pathways can be understood, and healthcare resources appropriately allocated.^34^ In this study, some Trusts explained that they did not have a clinical code in place to allocate to those with cancer-related pain. Almost a third of the 57 Trusts with a multi-disciplinary chronic pain service (n = 18) reported their service did not see people with cancer-related pain in response to a question about attendance rates. This is at odds with current Core Standards for Pain Management Services in the UK that recommend ‘People with cancer who may benefit from joint management with palliative care’ and ‘Cancer survivors’; that is, people with cancer who have undergone treatment (e.g. surgery, chemotherapy or radiotherapy) but who have chronic pain, should have access to specialist pain services.^14^ Such finding highlight the challenges healthcare professionals report identifying and referring to pain services^21,22^ and the difficulties patients have accessing such services.^18,19,35,36^

The lack of reporting and recording of demographic information, including gender, age and ethnicity, make it impossible to accurately describe or investigate possible inequity of access to pain services for those with cancer-related pain. Incomplete coding of ethnicity is an historical issue^37^ that impedes reliable analyses of ethnic differences and results in systematic biases in data quality.^38^ Scobie and colleagues (2021) found data quality problems affect records for global majority patients disproportionately. As there are significant differences in the provision of certain types of chronic pain services between areas of differing social deprivation in the UK,^39^ it is also vital that we understand the demographic information of attendees at pain services to understand equity of access.

It is recognised that diagnosing the specific type of cancer-related pain can be difficult and guidelines have been produced to help^11,40–43^; however, further guidance is needed to support healthcare professionals with this, as from a patient perspective, a diagnose is a key element of acceptance and validation.^44^ Recent recommendations to improve the experiences of cancer survivors living with chronic pain have highlighted the importance of diagnosing chronic pain in cancer survivors.^44^

Respondents reported that those with cancer-related pain were seen by cancer services, GPs, palliative care, community services, pain consultants or hospices. This suggests an assumption that there is a clear care pathway for these patients elsewhere in the health care system, but this is not reflected in the experiences of patients who feel they ‘bounce between services’ and find it hard to identify support^18^ and healthcare professionals who have difficulty in referring to services due to limited and inconsistent provision.^21,22^ This is further exemplified by findings from this study, as among the 97 Trusts that reported they do not have multi-disciplinary chronic pain service, nearly half advertised cancer services on their website (n = 44, 45%) and over a third advertised pain services (n = 36, 37%). Given this is public information on the internet, it is unsurprising that healthcare professionals, and the public, find it confusing and difficult to understand where services are available for those with cancer-related pain.

This study used FOI requests to access and collect data; achieving a response rate of 90% that included good coverage from across England. This could be attributed to the legal obligation organisations have to respond to FOI requests. However, the use of FOI mitigated against potential biases that respondents participated because they were specifically interested in cancer-related pain and were, therefore, keen to participate. Thus, this approach appears to have been helpful to gain a picture of access to multi-disciplinary chronic pain services for people with cancer-related pain across England. Organisations should respond to FOI requests within 20 working days,^28^ however, many Trusts took longer. The Freedom of Information Act^28^ allows for FOI requestors to submit a complaint if their request has not been responded to in a timely fashion. However, the researchers decided against this, because it was felt that Trusts may not have responded due to clinical pressures, and we did not want to put them under additional pressure. Organisations were sent a reminder about the FOI after 3 months and if there was still no response, they were not contacted again. During the stakeholder consultation for this study, the negative associations organisations can have with FOI requests was raised. This is consistent with reflections in the literature as Hammond suggests frontline clinicians may not welcome FOI requests for research purposes, and central to arguments opposing the use of FOI in this manner are cost and perceived lack of scientific value.^45^ To mitigate against such complaints, steps were taken to enhance trust between the researcher and organisations (such as the introductory email arising from the stakeholder consultation) which may have contributed to the high response rate.

### Limitations

The FOI request provided no opportunity for clarification, so it was vital to ask clear and unambiguous questions to gain accurate insights. To avoid ambiguity, we involved a wide range of stakeholders in the design of the FOI questions. However, there were some inconsistencies in the data collected, for example, large numbers of Trusts reported they did not have a multi-disciplinary chronic pain service, yet their website advertised such services. This may have been due to the wording of the questions and lack of definition of the term multi-disciplinary chronic pain service. It may also have been a reflection of the unclear and hazy picture of where people with cancer-related pain can access support and the challenge of identifying services.^18,21,22^ The inconsistencies may also be attributed to who completed the FOI, and their understanding of cancer-related pain. NHS Trust FOI departments determine who responds to FOI requests and in doing so make judgements about who is appropriate^45^ and researchers are not responsible for how an organisation responds to a request.^24^ Therefore, as we do not know if the requests were completed by an administrator or a clinician, it is hard to determine the responders’ knowledge of the nuances of cancer-related pain.

### Conclusions

Almost a third of multi-disciplinary chronic pain services in England reported not seeing people with cancer-related pain and, for services that do see people with cancer-related pain, few can provide attendance data. This highlights the challenges people living with cancer-related pain face accessing specialist pain provision and support. Further, a lack of a standardised method of coding and recording cancer-related pain as a reason for attending specialist pain clinics mean reporting accurate figures is challenging. It is essential that there is an accurate reporting system so the scale of cancer-related pain can be understood to help guide the provision and development of services, education and research.

## Supplemental material


Supplemental material - Mapping the gaps: Multi-disciplinary chronic pain service provision for cancer-related pain across England
Supplemental material for Mapping the gaps: Multi-disciplinary chronic pain service provision for cancer-related pain across England by Julie Armoogum, Alison Llewellyn, Fiona Cramp, Alice Berry, Candida McCabe in British Journal of Pain.
