# Supplementary material for: Mapping the gaps: Multi-disciplinary chronic pain service provision for cancer-related pain across England
Source: Br J Pain. 2026 Jul 17:20494637261465816. Online ahead of print. doi: 10.1177/20494637261465816 (PMC13380644; doi:10.1177/20494637261465816)
Supplement: Supplemental material - Mapping the gaps: Multi-disciplinary chronic pain service provision for cancer-related pain across England [file sj-pdf-1-bjp-10.1177_20494637261465816.pdf]

Supplementary file: FOI introductory email

Dear NHS Trust,

Hello, my name is Dr Julie Armoogum and I work at the University of the West of England in Bristol. We are trying to establish the provision of chronic pain clinics for people with cancer related chronic pain. We would be grateful if you could pass the attached Freedom of Information request questions to the most appropriate colleagues in the Trust. If you, or any of your colleagues, have any questions about this work, please do not hesitate to contact us. Many thanks for your time and consideration.

With best wishes,

Julie

Dr Julie Armoogum,

PhD, RN, RNT, NTF, SFHEA

Macmillan Senior Lecturer University of the West of England
